# A comprehensive insight into the correlation between ncRNAs and the Wnt/β-catenin signalling pathway in gastric cancer pathogenesis

**DOI:** 10.1186/s12964-023-01092-6

**Published:** 2023-06-29

**Authors:** Roozbeh Akhavanfar, Seyyed-Ghavam Shafagh, Behnood Mohammadpour, Yalda Farahmand, Mohammad Hassan Lotfalizadeh, Keihan Kookli, Ali Adili, Goli Siri, Seyed Mahmoud Eshagh Hosseini

**Affiliations:** 1 School of Medicine, Isfahan University of Medical Sciences, Isfahan, Iran; 2 Faculty of Medicine, Iran University of Medical Sciences, Tehran, Iran; 3 Medical School, Tonekabon Branch, Islamic Azad University, Tonekabon, Iran; 4 School of Medicine, Tehran University of Medical Sciences, Tehran, Iran; 5 School of Dentistry, North Khorasan University of Medical Sciences (NKUMS), Bojnurd, Iran; 6 International Campus, Iran University of Medical Sciences, Tehran, Iran; 7 Senior Adult Oncology Department, Moffitt Cancer Center, University of South Florida, Tampa, FL USA; 8 Department of Oncology, Tabriz University of Medical Sciences, Tabriz, Iran; 9 Department of Internal Medicine, Amir Alam Hospital, Tehran University of Medical Sciences, Tehran, Iran

**Keywords:** Gastric cancer, Non-coding RNA, miRNA, lncRNAs, Wnt/β-catenin signaling

## Abstract

During the past decades, gastric cancer (GC) has emerged as one of the most frequent malignancies with a growing rate of prevalence around the world. Despite considerable advances in therapeutic methods, the prognosis and management of patients with gastric cancer (GC) continue to be poor. As one of the candidate molecular targets in the treatment of many types of cancer, the Wnt/β-catenin pathway includes a family of proteins that have important functions in adult tissue homeostasis and embryonic development. The aberrant regulation of Wnt/β-catenin signaling is strongly correlated with the initiation and development of numerous cancers, including GC. Therefore, Wnt/β-catenin signaling has been identified as one of the main targets for extending therapeutic approaches for GC patients. Non-coding RNAs (ncRNAs), including microRNAs and long ncRNAs, are important components of epigenetic mechanisms in gene regulation. They play vital roles in various molecular and cellular processes and regulate many signaling pathways, such as Wnt/β-catenin pathways. Insights into these regulatory molecules involved in GC development may lead to the identification of potential targets for overcoming the limitations of current therapeutic approaches. Consequently, this review aimed to provide a comprehensive overview of ncRNAs interactions involved in Wnt/β-catenin pathway function in GC with diagnostic and therapeutic perspectives.

Video Abstract

**Supplementary Information:**

The online version contains supplementary material available at 10.1186/s12964-023-01092-6.

## Introduction

### Gastric cancer (GC)

Cancer, as the second foremost cause of death, is currently ranked the main public health problem affecting 19.3 million new cases and responsible for approximately 10.0 million deaths in 2020 [1]. Generally, more than 277 diverse types of cancer disease with different stages were identified indicating cancer pathogenesis is caused by several gene mutations. Due to accumulated abnormalities in numerous cellular regulatory arrangements, instead of proper reactions to the signals that regulate normal cell activities, cells constantly divide in an uncontrolled manner in a multi-stage procedure. Subsequently, without insensitivity to growth-inhibitory signals, mostly progress from a pre-cancerous lesion to invasive tumors that metastasize to distinct organs [2].

With 1.1 million new cases and nearly 1 million mortalities globally, GC is considered the 5th most prevalent malignancy and the 4th leading cause of cancer-related mortality, particularly among older males in 2020 [3]. According to the physical features, GCs are divided into two major sub-sites; it is thought that most of these cardia cancers arise in distal regions of the stomach (non-cardia) and the rest of the gastric cardia cancers (about 18%) are estimated to occur in the cardia, the part of the stomach that connects to the esophagus-stomach junction [4].

Although therapeutic approaches including adjuvant or neoadjuvant treatments combined with a surgical resection have helped to manage this disease, however, cancer invasion and metastasis result in a poor prognosis and low survival rate in GC patients. According to recent reports, the 5-year survival ranges from 60 to 80% for stage IA and IB tumors in patients undergoing surgery, and 18–50% for patients with stage III tumors undergoing surgery. These reports illustrate the urgency of an improved comprehension of molecular mechanisms participating in the initiation and progression of GC [5].

### Non-coding RNA

First, it was regarded that RNA plays a central role as an informational mediator between a gene coding sequence of DNA and its encoded protein. Most recently, based on the findings from the comprehensive genome sequencing, the total human genome has been recognized to contain less than 2% protein-coding, while over 90% of that is related to non-coding RNAs (ncRNAs). ncRNAs with no protein-coding role are constantly transcribed and extensively expressed in various organs and cells [6]. Previously, a large number of ncRNAs were regarded to be “junk RNAs”. However, during the last years, with the progress of research in the field of molecular biology, it has illustrated that ncRNAs have fundamental roles and prevent the translation activity of mRNA, control the target genes expression, adjust biological signaling pathways, and control cell fates. Hence, the mutation or deregulation of ncRNAs may affect the initiation and development of diseases, including cancer [7]. According to the length of their sequence, lncRNAs are divided into three groups: (1) short ncRNAs with shorter than 50 nucleotides (nt), including microRNAs (miRNAs), small interfering RNAs (siRNAs) and Piwi-interacting RNAs (piRNAs); (2) mid-size ncRNAs between 50 and 200 nt; and (3) long non-coding RNAs (lncRNAs) are longer than 200 nt. Also, ncRNAs are categorized into two types, housekeeping ncRNA, containing transfer RNAs (tRNAs) and highly abundant ribosomal RNAs (rRNAs) with a vital role in basic cell function, have large quantities, and are ubiquitous in cells. Regulatory ncRNAs, as another type, include miRNAs, lncRNAs, piRNAs, circular RNAs (circRNAs), small nucleolar RNA (snoRNAs), tRNA-derived small RNA (tRFs), siRNAs, and so on. These ncRNAs are considered as central regulatory RNA molecules that participate in regulatory mechanisms of gene expression, containing epigenetic, transcriptional, and post-transcriptional levels [8]. Among the most studied classes of ncRNAs, miRNAs and lncRNAs are introduced.

As most characterized ncRNAs, miRNAs are short sequences of RNA with about 19–24 nt length. MiRNAs are highly conserved and single-stranded modulating diverse biological activities, including cellular differentiation, proliferation, apoptosis, invasion, and metastasis [9]. MiRNA partially or completely binds to mRNA sequence and, respectively, prevents the translation of protein or causes mRNA degradation. Interestingly, one miRNA could simultaneously control numerous target mRNAs and subsequent signaling pathways. Due to their role in cellular activities, dysregulation of miRNA expression is correlated with the initiation and development of many types of cancer [10]. Hence, miRNAs turn into the main topic of clinical research in terms of their diagnostic and therapeutic potential for various malignancies.

LncRNAs are RNA molecules with a length of more than 200 nucleotides, that rarely indicate the protein-coding capacity [11] and play critical roles in transcriptional, post-transcriptional, and epigenetic regulation of gene expression and function [12]. It is reported that lncRNAs are transcribed and synthesized in a similar manner as mRNAs [13]. Also, compositional analysis and structural characteristics of lncRNAs illustrated that lncRNAs share some similarities with protein-coding RNAs in terms of tissue and time-specific expression [14]. They have vital physiological and biochemical roles in growth, differentiation, and X-inactivation [15] and play a critical role in gene imprinting, transcriptional activation, chromatin modification, splicing, translation regulation, and cell cycle regulation [16–18]. Functional elements of lncRNAs are divided into two types, including interactor elements and structural elements. Former refers to a part of lncRNA that is capable of forming direct physical interaction with other molecules, such as proteins and DNAs. Latter refers to the elements within lncRNA that lead to the formation of the secondary and/or tertiary structures controlling their functional interactions [19]. The reason that lncRNAs function in various ways is their capacity to interact with other molecules by base pairing or chemical interactions in secondary structures [20, 21]. Recently, lncRNAs have obtained significant attention due to their key roles in numerous biological processes. Several researchers discovered that dysregulated lncRNAs were related to disease initiation, development, proliferation, metastasis, and invasion [22, 23]. Therefore, a fundamental investigation of lncRNA functions in cancer diseases can help to improve therapeutic approaches.

### Wnt signaling pathway

According to recent reports, large amounts of signaling pathways are involved in the pathogenesis of many types of malignancies. With the considerable improvement in the comprehensive analysis of genome structure in tumorigenesis using advanced sequencing technologies, it was identified that Wnt signaling pathway is one of the key oncogenic signaling cascades that is tightly associated with cancer pathogenesis and plays a vital role in cancer development and metastasis. It preserves genetic stability and has a central role in the determination of cell fate, cell proliferation, differentiation, motility, and apoptosis as well as the maintenance of stemness features [24]. The Wingless-type MMTV integration site family members, shortly known as WNTs, contain 19 glycoproteins enriched by cysteine residues [25] with post-translational alterations, including palmitoylation and glycosylation crucial for their biological activity. The interactions of WNTs with their particular receptors stimulate WNT pathways regulating vital cellular processes. [26]. This signaling pathway is mainly categorized into two different types, including β-catenin dependent pathway, known as canonical pathway, and β-catenin independent pathway, named non-canonical signaling. The canonical pathway is triggered by the interaction of Wnt ligands, including Wnt1 and Wnt3a, with a Frizzled (Fz) receptor and Lipoprotein Receptor-Related Protein (LRP) 5/LRP6. Then, Fz interacts with and activates a cytoplasmic protein, known as Disheveled (DVL in mammals), leading to the accumulation of β-catenin, a vital signal transducer of canonical pathway, in the cytoplasm and its translocation into the nucleus and target gene transcription. This is achieved by the DVL-mediated suppression of the β-catenin-destruction complex. In unstimulated conditions, β-catenin cytosolic phosphorylation and degradation are essential for the regulation of canonical Wnt signaling. The modulation of β-catenin phosphorylation-mediated proteolysis is controlled by the β-catenin-destruction complex consisting of casein kinase 1 (CK1), glycogen synthase kinase 3α/β (GSK-3α/β), Axis inhibition protein 1 (AXIN1) and adenomatous polyposis coli (APC) [27]. In this complex, GSK3β and β-Transducin Repeat-Containing Protein (β-TrCP), an E3 ubiquitin ligase, downregulate β-catenin through its phosphorylation and ubiquitination, respectively [28]. Consequently, β-catenin is targeted to the proteasome for the degradation process. Therefore, by the absence of β-catenin in the nucleus, an inhibitory complex comprising transducing-like enhancer protein (TLE/Groucho) and T-cell factor/lymphoid enhancer-binding factor (TCF/LEF) employs histone deacetylases (HDACs) to suppress the expression of β-catenin target genes [29]. By the way, in a stimulating situation, stabilized β-catenin is translocated into the nucleus and creates an active complex in cooperation with TCF/LEF through displacing Groucho/TLE complexes and employment of histone modifying co-activators (e.g. CBP/p300, Pygo, BCL9, and BRG1[30]. By switching this transcriptional activity, Wnt/β-catenin activation causes changes in various cellular processes, including cell proliferation differentiation and stemness [24, 31]. Some studies have reported that various Genetic mutations can deactivate the β-catenin-destruction complex or upregulate β-catenin expression, resulting in the transactivation of Wnt target genes by TCF4/β-catenin complexes [32, 33].

**Fig. 1:**
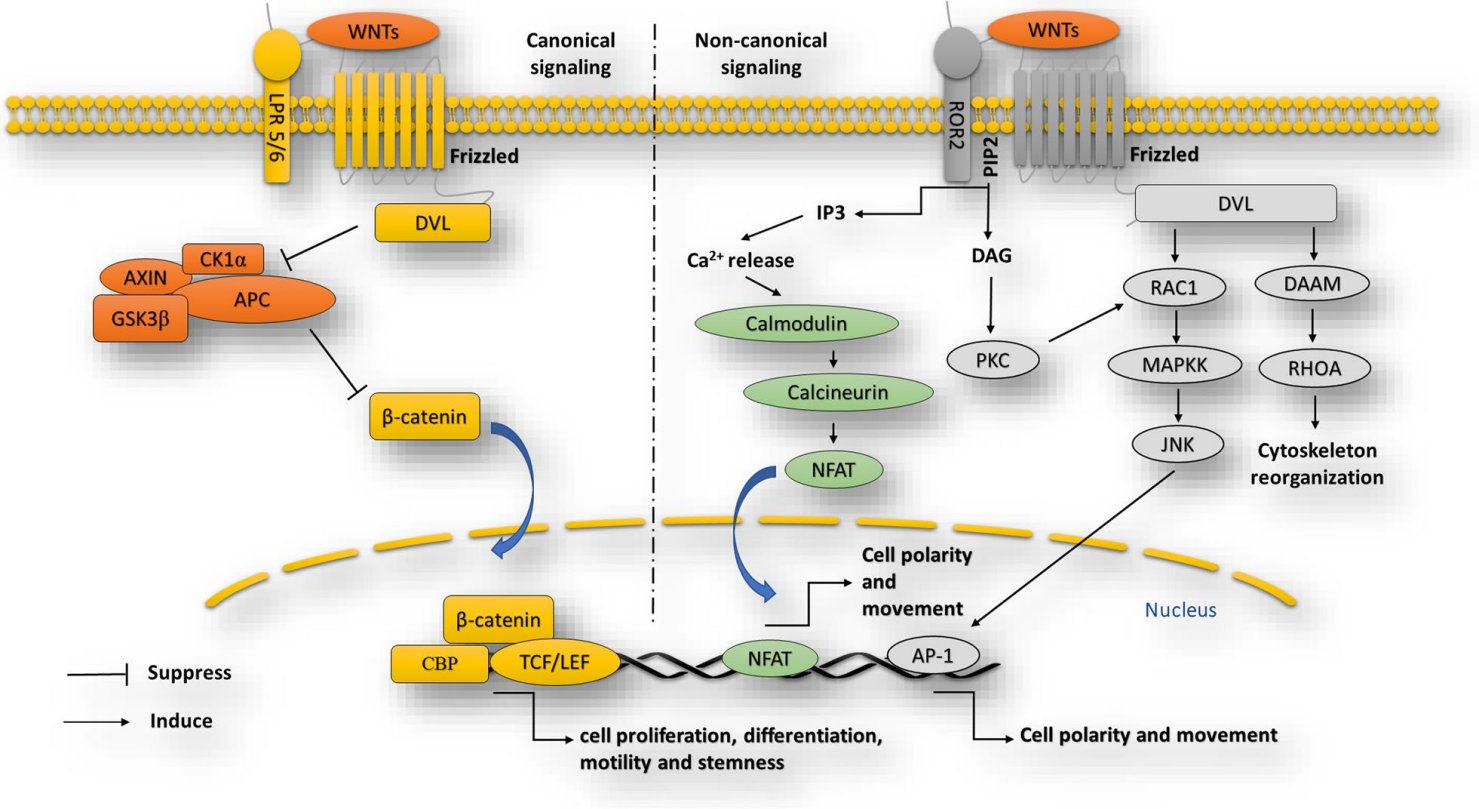
The canonical and non-canonical Wnt signaling pathways. The canonical pathway is characterized by the stabilization and intracellular accumulation of β-catenin through the deactivation of β-catenin-destruction complex (APC, AXIN, GSK3, and CK1α) mediated by DVL proteins. In this pathway, β-catenin is translocated into the nucleus and by forming a complex with other elements, such as CBF, activates the expression of target genes. However, the non-canonical Wnt signaling is a β-catenin-independent pathway. This pathway is triggered by the binding of Wnt ligands to the ROR-Frizzled receptor complex, leading to the activation of DVL. In turn, DVL protein activates Rho small GTPase by the de-inhibition of cytoplasmic protein DAAM, and stimulates Rac1 signaling. Rho and Rac1 mediate the activation of ROCK and JNK and promote polarized cell migration (PCP signaling). Besides, non-canonical Wnt signaling leads to the production of DAG and IP3 by PLC activity. DAG activates PKC signaling, and IP3 production stimulates Ca^+2^ pathway (also provokes PKC signaling) and NFAT-dependent transcriptional response

The β-catenin-independent pathway does not include β-catenin–TCF or β-catenin–LEF components but utilizes alternative means of downstream signaling, which may stimulate a transcriptional response. The non-canonical Wnt signaling, participating in cell polarity and migration, is further divided into WNT/planar cell polarity (PCP) and calcium (Ca^+2^) pathway. These pathways antagonize WNT/β-catenin-signaling in some conditions [27, 34]. In Wnt/PCP signaling, Wnt ligands bind to the Retinoic acid-related Orphan Receptors (ROR)-Frizzled receptor complex, leading to DVL activation [35]. Then, through the activity of the cytoplasmic protein DVL-associated activator of morphogenesis 1 (DAAM1), DVL binds to the small GTPase Rho [36]. Rho kinase (ROCK) and c-Jun NH2-terminal kinases (JNKs) are activated by the corporation of the small GTPase Rac1 and Rho that results in reorganizations of the cytoskeleton and/or transcriptional activities [37]. On the other hand, in Wnt/Ca^2+^ cascade, the binding of Wnt ligand to a Fz receptor activates phospholipase C (PLC), located on the plasma membrane of the cell [38]. PLC activity leads to the production of specific signaling molecules, including diacylglycerol (DAG) and 1,4,5-triphosphate (IP3). IP3 causes the intracellular release of Ca^+2^ ions which in turn activates effector molecules such as calmodulin-dependent kinase II (CAMKII), calcineurin, and protein kinase C (PKC). These events eventually activate the transcriptional regulator NFAT (nuclear factor of activated T cells), resulting in calcium-dependent cytoskeletal reorganization and/or transcriptional responses (Fig. 1) [39].

Regarding the mechanism of Wnt signaling cascade, it participates in several biological activities, such as development, cellular growth, differentiation, tissue regeneration, organogenesis, and homeostasis [40]. A dysregulated Wnt pathway can lead to many types of diseases including cancer [24]. Hence, a better understanding of the molecular mechanisms which regulate Wnt signaling cascade in various types of cancer, such as GC, is important for the development of suitable treatment approaches. One of the associated molecular mechanisms for regulating Wnt pathway comprises ncRNAs such as miRNA and lncRNAs. According to previous reports, ncRNAs may have a crucial function in upstream signals and are recognized to contribute to the pathogenesis of GC by activating or preventing the Wnt signaling pathway. Moreover, Wnt may transcriptionally modulate the expression and the activity of numerous ncRNAs in several types of human cancers, including GC. To provide novel approaches for Wnt-targeted cancer therapy, summarizing the ncRNAs associated with the Wnt-mediated signaling pathway in GC is required.

## Wnt/β-catenin pathway and GC

Genome and transcriptome analysis evidenced that about 46% of GC patients demonstrate the deregulated Wnt/β-catenin pathway. According to growing evidence, numerous mutated components of canonical Wnt signaling, which further lead to abnormal activity of this pathway, have a dominant function in malignant transformation and GC invasiveness. These abnormalities in GC can be related to Wnt ligands, cytoplasmic Wnt components, Wnt target genes, Wnt antagonists, and Wnt receptors. Numerous Wnt ligands are overexpressed in human GC, such as Wnt-1, Wnt-2b, Wnt-6, and Wnt-10a,b [41]. Reports indicated that upregulated Wnt-1 ligand can either maintain the GCSC stemness or promote the advanced stages of GC [42]. Furthermore, overexpression of Wnt-2 leads to β-catenin cytoplasmic accumulation and nuclear localization in intestinal and diffuse-type GC, which is related to malignant properties of GC, such as tumor invasion or migration [43]. Regarding the reports of a study, axis duplication in 90% of embryos was induced through ventral injection of synthetic Wnt-2b2 mRNA. These findings tightly indicate that overexpression of Wnt2b2 in some cases of GC may result in tumor progression by upregulating β-catenin–TCF signaling pathway [44]. Also, in GC patients, Wnt-6 expression was positively associated with the nodal status and the tumor stage. Wnt-6 is inversely associated with the response after chemotherapy and increases the resistance to anthracycline drugs in GC cells [45]. Additionally, Wnt10B has been shown to function as an oncogene that triggers GC development [46]. Other constituents of Wnt/β-catenin pathway include cytoplasmic Wnt components, including APC, β-catenin, AXIN2, and Gsk3β. The functional deactivation of APC is often because of gene mutation or hyper-methylation of its promoter region. The results of a study indicated that 9 /16 of high-grade GC patients had a methylated APC promoter that related to dysplasia grade and aberrant β-catenin expression. This hyper-methylation in APC promoter can have a central function in the tumor initiation and progression in GC via the stimulation of Wnt signaling [47]. Additionally, whole-genome profiling categorizes the Wnt pathway as the third most functioning signaling in GC through its frequently mutated constituents, Catenin Beta 1 (CTNNB1), Ring Finger Protein 43 (RNF43), AXIN1/2 and APC [48]. Further, it has been revealed that about 30% of microsatellite instability indicates AXIN2 frameshift mutation [49]. In a previous study, the expression of β-catenin and Axin 2, cyclinD1, and c-myc were low expressed/overexpressed in mRNA and protein levels through the inactivation/stimulation of the Wnt cascade, respectively [50]. In addition, it is reported that mutated exon 3 of β-catenin are frequent in GC that demonstrate nuclear β-catenin [51]. Also, as another abnormality in Wnt signaling components, genetic deletion of Gsk3β, drives rapid GC formation in mice [52]. Currently, one of the abnormalities related to Wnt components was identified in the level of the ligand/receptor in several cancers, especially in GC. Their frequency is higher than the deregulation of cytoplasmic components [53]. Many studies have evaluated the Wnt antagonist and its function in controlling this signal. According to a previous study, promoter methylation of Dickkopf WNT signaling pathway inhibitor (DKK1/2) and Secreted Frizzled Related Protein (SFRP2/4) was considerably increased in GC. Furthermore, simultaneous hyper-methylated SFRP2 and KK2 were detected in GC which shows a significant relation with overexpression of β-catenin. This event causes the activation of Wnt pathway by the joint inactivation of these two genes [54]. Also, Hong et al. indicated an increased level of DKK1 expression in tumor samples showing lymph node metastasis [55]. Frizzled-7 (Fzd7), as the predominant Wnt receptor has been shown to increase in human GC cells, which correlates with poor clinical outcomes in patients with GC. It is demonstrated that Fzd receptors, in particular Fzd7, are rate-limiting for the proliferation of GC cells with or without mutated APC in-vivo. Also, pharmacologic suppression of Fzd repressed the GC proliferation [56]. To sum up this section, Wnt signaling involved in the initiation, growth, and progression of GC, is highlighted as a promising target for GC precision therapy.

## The regulation of Wnt/β-catenin signaling by ncRNAs in GC

### MiRNAs directly target Wnt/β-catenin signaling in GC

Currently, the interaction between miRNAs and Wnt/ β-catenin signaling pathways as well as their roles in many types of cancers such as GC was evaluated. It is manifested that Wnt/β-catenin signal and miRNAs are important modulators of development and almost all Wnt signaling components are controlled by miRNAs. Besides, the balance between the activity of miRNAs and Wnt cascades is accomplished through direct and indirect monitoring mechanisms which can lead to cellular homeostasis. Therefore, abnormalities in Wnt signaling pathways and miRNA levels cause an aberrant developmental process that can cause many types of cancer, such as GC. Hence, a deep understanding of miRNA roles in regulating Wnt/β-catenin cascade would be beneficial for the development of novel therapeutic methods for GC [57].

**Fig. 2:**
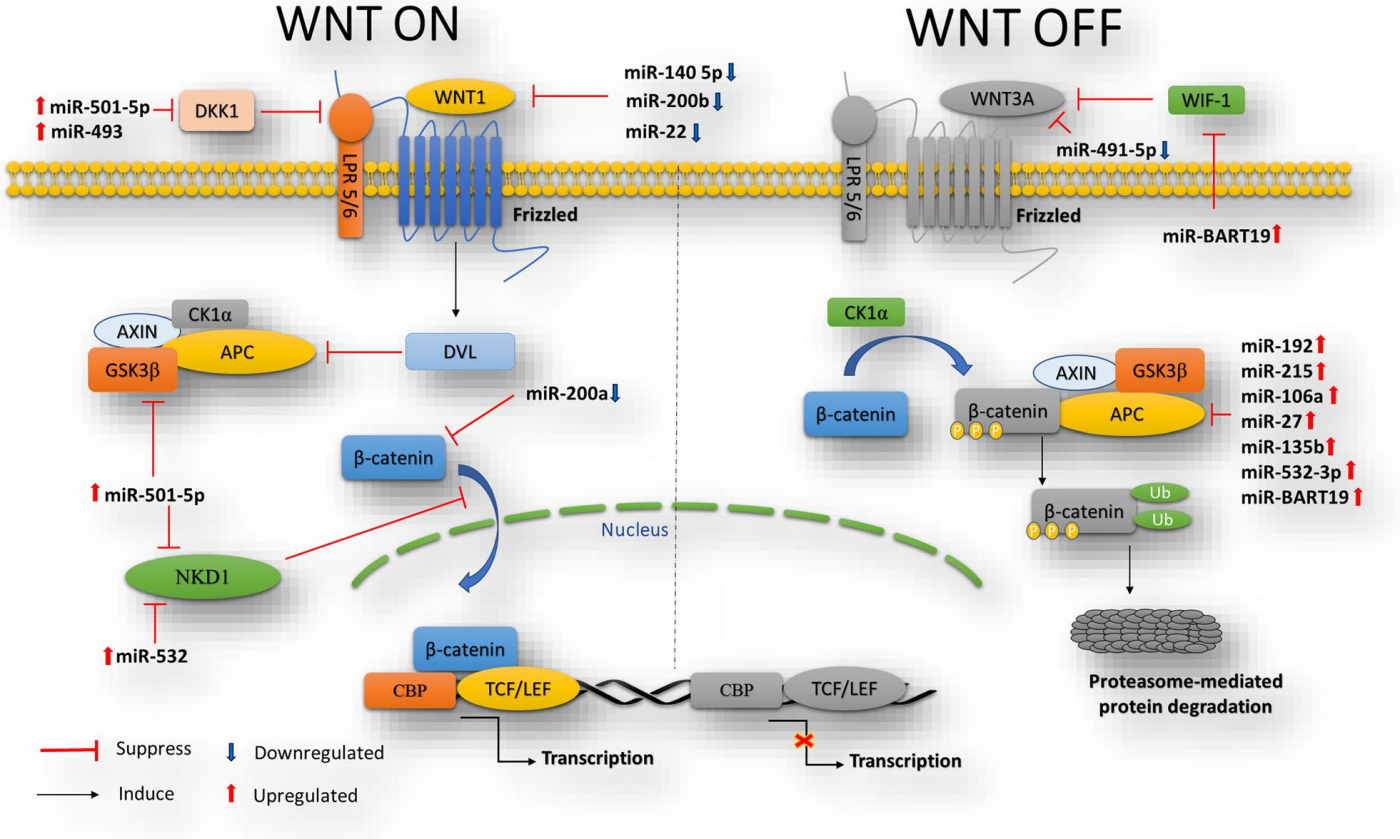
miRNAs directly regulate the components of canonical Wnt signaling in GC

Several miRNAs were reported to directly regulates Wnt/β-catenin signaling pathway by targeting Wnt components in GC (Fig. 2). These miRNAs either function as a oncomiR and target the main suppressors of Wnt/β-catenin signaling, especially APC, or have a tumor suppressive role by targeting vital mediators of this pathway, such as β-catenin.

As an oncogenic miRNA, miR-501-5p was revealed to be significantly overexpressed in GC cell lines and tissues and to increase the stem cell-like features in GC cells. Upregulated miR-501-5p led to hyper-activity of Wnt/β-catenin signaling by directly suppressing several repressors of this pathway, namely DKK1, GSK3β, and Naked cuticle 1 (NKD1). DKK1 is known to be a secreted protein that functions as a negative regulator of Wnt signaling that binds to and antagonizes the function of LRP6 [58]. NKD1 is also a negative regulator of the canonical Wnt signaling, which is downregulated in gastric tumorigenesis and has a suppressive effect on Dishevelled-1 and β-catenin protein levels. NKD1 suppression leads to Wnt signaling mediated tumor invasion through upregulating MMP-7 [59]. Besides, miR-493 has been reported to be highly expressed in GC, participating in tumorigenesis. This miRNA also directly targeted and decreased the DKK1 expression and increased proliferation, invasion, and chemo-resistance of GC cells [60]. Additionally, it is found that miR-532, which is upregulated in GC tissues and cells, promoted GC cellular migration and invasion through direct suppression of NKD1 expression and inducing Wnt/β-catenin pathway activity [61]. Besides the NKD1 and DKK1, some oncomiRs exert their promoting effect on Wnt/β-catenin pathway by targeting another repressor of this pathway, namely APC. Among these oncomiRs, miRNA-192 and -215 have been shown to participate in GC cellular proliferation and migration ability by directly targeting APC, as a part of β-catenin-destruction complex, which is also downregulated through gastric tumorigenesis [62]. Furthermore, miR-106a-3p showing high expression levels in GC tissues in comparison with normal samples was demonstrated to directly target APC mRNA. miR-106a-3p suppression repressed EMT and metastasis by deactivating Wnt/β-catenin cascade by rescuing APC expression [63]. Another research illustrated that miR-27 oncogene provoked EMT process by triggering Wnt pathway mediated by the suppression of APC expression. APC was recognized as the direct and functional target of miR-27 [64]. According to a previous study, APC suppression caused by miR-135b-5p overexpression resulted in higher transduction of β-catenin signaling, and subsequently a greater proliferative ability of GC cells [65]. Zhao et al. indicated that not only the promoters of Wnt inhibitory factor 1 (WIF1), APC, and NLK are highly methylated in nasopharyngeal carcinoma and GC cell lines, but also WIF1 and APC are also targeted and downregulated by EBV miR-BART19-3p, leading to an increase in the cellular proliferation. Therefore, it was suggested that Wnt pathway activity may be boosted via miR-BART19-3p in Epstein-Barr virus (EBV)-related tumors [66].

Besides the oncomiRs, some studies have proven that tumor suppressor miRNAs are also involved in the regulation of Wnt/β-catenin signaling through gastric tumorigenesis. As mentioned in the following, these miRNAs mainly target Wnt ligands and β-catenin. As an important example, a negative correlation was found between the expression of miR-200a, a known tumor suppressor in GC, and the activity of β-catenin pathway. The findings indicated that miR-200a directly targeted β-catenin mRNA and hindered gastric tumorigenesis by suppressing the pathway [67]. Additionally, as mentioned before, Wnt ligands can regulate by miRNAs. It was indicated that miR-140-5p expression, as a tumor suppressor, was considerably reduced in GC tissue samples compared to adjacent non-tumor samples, showing a correlation with lymph node metastasis in GC patients. The results of bioinformatic analysis and dual-luciferase assays revealed that miR-140-5p directly targets and downregulates WNT1 ligand. Decreasing miR-140-5p expression considerably promoted cellular proliferation and invasion capability, as well as increased the activity of Wnt/β-catenin signaling through increasing β-catenin and WNT1 expression in GC [68]. Likewise, Wnt-1 was recognized as a target of both miR-22 and miR-200b which act as tumor suppressors. Both of them prevent Wnt/β-catenin signaling through direct targeting Wnt-1 in GC cells [69]. Furthermore, miR-491-5p, as another tumor suppressor miRNA, which showed low expression levels in GC, was evidenced to target Wnt3a ligand and prevent GC cell proliferation by stimulating apoptosis [70].

Taken together, these findings illustrate the importance of miRNAs as alternative targets in the regulation of Wnt/β catenin signaling to be considered for targeted therapy of GC (Table 1).

**Table 1:** MiRNAs directly target Wnt/β-catenin signaling pathway in GC

MicroRNA	Oncogene/suppressor	Biological functions	Target genes	References
miR-501-5p	Oncogene	Stem cell-like phenotype	DKK1, GSK3β, and NKD1	[58]
miR-493	Oncogene	Proliferation, invasion, and chemo-resistance	DKK1	[60]
miR-532	Oncogene	Migration and invasion	NKD1	[61]
miR-140-5p	Suppressor	Proliferation, invasion	WNT1	[68]
miR-200b	Suppressor	Proliferation, apoptosis	WNT1	[69]
miR-22				
miR-491-5p	Suppressor	Proliferation, colony formation, apoptosis	Wnt3a	[70]
miR-192	Oncogene	Proliferation, migration	APC	[62]
miR-215				
miR-106a-3p	Oncogene	EMT and metastasis	APC	[63]
miR-27	Oncogene	EMT	APC	[64]
miR-135b-5p	Oncogene	Proliferation	APC	[65]
miR-BART19-3p	Oncogene	Proliferation	WIF1, APC	[66]
miR-532-3p	Oncogene	Proliferation, EMT, migration	APC	[71]
miR-200a	Suppressor	Viability, invasion, EMT	β-catenin	[67]

### MiRNAs that modulate Wnt/β-catenin signaling pathway via mediators in GC

Some miRNAs have been reported to modulate Wnt/β-catenin signaling by regulating other mediators which are not the main components of the pathway. The suppressor of fused homolog (SUFU), a negative regulator of Ηedgehog signaling, is targeted by various miRNAs and plays important role in the regulation of Wnt/β-catenin signaling. According to a recent study, miRNA-194 was upregulated in GC tissues while SUFU indicated low expression levels in GC. The inhibition of miR-194 expression, attenuated β-catenin nuclear accumulation by rescuing SUFU expression, and blocked Wnt/β-catenin signaling, leading to a decrease in malignant features [72]. Also, miRNA-324-5p was previously shown to be overexpressed in GC and stimulate Wnt signaling by regulating SUFU expression [73]. miRNA-20b is another tumor suppressor miRNA involved in SUFU-mediated regulation of Wnt/β-catenin signaling. It was demonstrated that miRNA-20b directly targets SUFU and activates Wnt pathway, which in turn induces GC cell proliferation, EMT, migration, and colony formation [74]. Additionally, it is discovered that miRNA-150, as an oncogene, directly targets SUFU to stimulate proliferation, EMT, and migration, via the dual activation of Wnt and Hedgehog signaling pathways [75].

By the way, other oncogenic miRNAs induce Wnt/ β-catenin cascade by regulating a wide array of molecular components rather than SUFU. A study reported that miR-188-5p reduces the expression of Phosphatase and tensin homolog (PTEN) as a tumor suppressor, and increases phospho-Ser9 of GSK3β to trigger Wnt/β-catenin cascade in GC. This leads to an increase in GC cell invasion and migration. In summary, a model of the miR-188-5p-PTEN-β-catenin axis, which facilitates the stimulation of Wnt/β-catenin signaling and triggers metastasis ability, was reported [76]. Also, miR-199a-5p was reported to function as an oncomiR that suppresses E-cadherin expression and induces EMT and metastasis in GC through stimulating nuclear translocation of β-catenin [77]. SMG1 (SMG1 Nonsense Mediated MRNA Decay Associated PI3K Related Kinase) (SMG-1), as a tumor suppressor, was significantly downregulated in GC tissues. The suppression of SMG-1 by miR-192 and miR-215 oncomiRS also reduced the activity of Wnt signaling and prompted EMT in GC cells [78]. Besides, miR-675 upregulation, as a predictor of worse prognosis in patients with GC, particularly stimulated cellular proliferation, invasion, and migration in AGS cells. PITX1 (Paired Like Homeodomain 1), as a tumor suppressor, was proven to be a direct target of miR-675, and its downregulation led to EMT stimulation through Wnt/β-catenin signaling[79]. Additionally, miRNA-27a was also reported to stimulate the proliferation and invasion of MGC803 cells by targeting SFRP1 (Secreted Frizzled Related Protein 1) and modulating Wnt/β-catenin signaling. miRNA-27a suppression resulted in SFRP1-mediated downregulation of β-catenin, Wnt, p-β-catenin, and p-Wnt [80]. It was, moreover, demonstrated that miR-544a, which induces EMT phenotype in GC, could target Cadherin 1 (CDH1) and AXIN2 which are involved in the degradation and the translocation of β-catenin. miR-544a-mediated suppression of CDH1 and AXIN2 stabilized β-catenin expression and increased its nuclear translocation, leading to Wnt signaling activation and tumor progression [81].MiR-93-5p is another oncomiR that targets and negatively regulates AHNAK (Neuroblast differentiation-associated protein), a tumor suppressor, leading to GC cell migration and invasion via the activation of Wnt signaling in GC [82]. SMAD4 (SMAD Family Member 4) is a tumor suppressor that is downregulated through gastric tumorigenesis. SMAD4 also participates in the negative regulation of Wnt signaling. A study showed that miR-324-3p with a high expression in GC, stimulated growth, migration, and reduced apoptosis ability by targeting Smad4 and activating Wnt/beta-catenin signaling pathway [83].

On the contrary, tumor suppressor miRNAs have been reported to target various oncogenes participating in positive regulation of Wnt/β-catenin signaling. As an example, miR-154 has been shown to suppress the proliferation and invasion of GC cells by directly targeting the Dishevelled–Axin domain containing 1 (DIXDC1), a positive regulator of Wnt/beta-catenin signaling. Further investigations indicated that restoration of DIXDC1 expression notably hindered the inhibitory effect of miR-154 on cellular growth, invasion, and WNT signaling in GC cells [84]. Furthermore, miR-338, as a tumor suppressor, prevented cellular proliferation, invasion, and migration ability in GC cells by enhancing the phosphorylation of GSK-3βSer9 and downregulating c-Myc protein expression, leading to the deactivation of Wnt/β-catenin signaling Mechanistically, Also, miR-338 inhibited the expression ephrin type-A receptor 2 (EphA2) and led to EMT suppression by E-cadherin overexpression and Vimentin and β-catenin downregulation[85] EphA2 oncogene acts as a receptor for Wnt ligands and by forming a complex with Dvl2/Axin1 suppresses β-catenin-destruction complex, inducing β-catenin nuclear translocation and c-MYC transcription [86]. miR-302b was also reported to directly target and downregulate EphA2 in GC cells, decreasing cellular migration, invasion, and cellular growth through modulating β-catenin signaling [87]. Furthermore, it was demonstrated that miR-503 expression level was notably lower in GC tissues and cells in comparison with normal gastric samples; showing a correlation with lymph node metastasis and tumor size. miR-503 overexpression suppressed proliferation, cell invasion, and colony formation in GC cells by targeting HMGA2 (High Mobility Group AT-Hook 2) transcription factor. Also, miR-503-mediated HMGA2 downregulation repressed WNT/β-catenin cascade in GC cells by increasing the phosphorylation of β-catenin and GSK-3β expression, and reducing β-catenin expression and p-GSK-3β phosphorylation [88]. miR-330-3p was downregulated, whereas PRRX1 (Paired Related Homeobox 1) was upregulated in the GC patients’ serum. Moreover, activation of miR-330-3p and inactivation of PRRX1 prevented the expression levels of β-catenin, p-GSK-3β, N-cadherin, cyclin D1, and vimentin proteins, while increasing p-β-catenin, GSK-3β, and E-cadherin protein expressions. Also, miR-330-3p was suggested to suppress cellular proliferation, EMT, and invasion and increase apoptosis rate by hampering PRRX1-mediated Wnt/β-catenin cascade in GC [89]. In another study, miR-511 with a low-expression level indicated an inverse association with TRIM24 (Tripartite Motif Containing 24) in primary GC tissues and cell lines. The upregulation of miR-511 prevented proliferation, cell cycle progression, and colony formation ability as well as inactivated Wnt/β-catenin and PI3K/AKT pathways by directly targeting TRIM24 oncogene in GC cells [90]. Zhou w et al. indicated that miR-6838-5p was downregulated in GC cells and its upregulation suppressed proliferation, cell cycle progression, migration, and invasion. In a mechanistic view, miR-6838-5p, as a tumor suppressor, prevented GC cell malignant behaviors by regulating GPRIN3 (GPRIN Family Member 3) expression and blocking Wnt/β-catenin pathway [91]. The results of a study demonstrated that miR-507 was considerably downregulated in GC tissues and cell lines, and upregulated miR-507 notably prevented growth and invasion and induced apoptosis in GC cells. Chromobox 4 (CBX4), as a downstream target of miR-507, could reverse the function of miR-507 on the GC cells. It was confirmed that miR-507 could prevent the expression of CBX4 to block the activation of Wnt/β-catenin and Hypoxia-Inducible Factor (HIF)-1α pathways [92]. In a molecular survey, it is reported that decreasing or deleting E-cadherin leads to the flow of the β-catenin from cadherin/catenin complexes. The upregulated miR-200a improved the expression of E-cadherin and diminished the Wnt/β-catenin cascade by targeting ZEB1 and ZEB2 in GC cells [93]. miR-29c-3p is another tumor suppressor miRNA involved in Wnt/β-catenin cascade that directly targets KIAA1199 oncogene, an inducer of cell migration. KIAA1199 suppression resulted in a decrease in the expression of some main proteins in Wnt/β-catenin and epidermal growth factor receptor (EGFR) signaling pathways (e.g., FGFR4, WBP11 and PTP4A3). Hence, miR-29c-3p was suggested to control GC cellular migration by modulating the expression of KIAA1199 and stimulating EGFR and the FGFR4/Wnt/β-catenin signaling pathways [94]. Another study revealed that miR-381 and miR-489, which are downregulated through gastric tumorigenesis, directly target Cullin 4B (CUL4B), as a tumor-promoting gene, prevents GC cellular growth, migration and invasion through blocking the Wnt/β-catenin pathway. CUL4B activates Wnt/β-catenin signaling by rescuing β-catenin from GSK3-mediated degradation and epigenetic silencing of Wnt pathway antagonists, such as DKK1 [95, 96]. The results of recent research indicated that reduced expression of YWHAZ (Tyrosine 3-Monooxygenase/Tryptophan 5-Monooxygenase Activation Protein Zeta) repressed EMT, migration, and invasion of GC cells. YWHAZ was verified to be a direct target of miR-375 tumor suppressor. Furthermore, miR-375-mediated YWHAZ suppression hindered the activation of Wnt/β-catenin cascade. Hence, the miR-375 inhibitor stimulated GC cell metastasis features by activation of Wnt/β-catenin pathway in GC cells [97]. Additionally, miR-338-3p, as a tumor suppressor, prevented cellular proliferation and induced apoptosis in GC cells by downregulating Wnt/β-catenin signaling. miR-338-3p was shown to negatively modulate the expression of SOX5 (SRY-Box Transcription Factor 5), an inducer of Wnt/β-catenin cascade, by directly binding to its mRNA sequence. Moreover, miR-338-3p-mediated blockage of Wnt/β-catenin signaling was significantly abolished via upregulated SOX5 [98], showing the significance of miR-338-3p in targeting SOX5-mediated Wnt/β-catenin signaling in GC. Also, miRNA-219-5p was reported to suppress gastric tumorigenesis mediated by Wnt/β-catenin signaling through targeting another positive regulator of this pathway, Liver receptor homolog 1 (LRH-1) [99]. Besides, CYR61 (cysteine-rich angiogenic inducer 61, CCN1), another oncogene stimulating Wnt/β-catenin pathway, has been reported to be targeted and downregulated by miR-142-5p, an anti-tumor miRNA. MiR-142-5p decreased GC cell migration and invasion by suppressing CYR61-mediated Wnt/β-catenin cascade [100]. Yes-associated protein (YAP) is one of major components of Hippo signaling that has been shown to also stimulate Wnt/β-catenin signaling. The overexpression of miR-195-5p, which is downregulated in GC, inhibited YAP expression and prevented GC cell proliferation and invasion as well as increased cell apoptosis. miR-195-5p-mediated downregulation YAP also led to the downregulation of β-catenin, cyclin D1, and c-myc protein and stimulate the expression of Caspase-3/9 proteins. Then, miR-195-5p could be suggested an important target in moudalting YAP-mediated Wnt/β-catenin signaling in GC [101] (Table 2).

**Table 2:** MiRNAs that modulate the Wnt/β-catenin signaling pathway via mediators in GC

MicroRNA	Oncogene/suppressor	Biological functions	Target genes	References
miRNA-194	Oncogene	Apoptosis	SUFU	[72]
miRNA-324-5p	Oncogene	Growth, migration, EMT	SUFU	[73]
miR-188-5p	Oncogene	Invasion, migration	PTEN	[76]
miR-154	Suppressor	Proliferation, invasion	DIXDC1	[84]
miR-503	Suppressor	Proliferation, cell invasion, and colony formation	EphA2	[88]
miR-338	Suppressor	Proliferation, migration invasion	HMGA2	[85]
miR-330-3p	Suppressor	Proliferation, apoptosis, EMT, and invasion	PRRX1	[89]
miR-6838-5p	Suppressor	Proliferation, cycle progression, migration, invasion	GPRIN3	[91]
miR-302b	Suppressor	Migration, invasion	EphA2	[87]
miR-507	Suppressor	Growth, invasion apoptosis	CBX4	[92]
miR-200a	Suppressor	Growth, EMT	ZEB1/2	[93]
miR-199a-5p	Oncogene	EMT	E-cadherin	[77]
miRNA-20b	Oncogene	Proliferation, EMT, migration colony formation	SUFU	[74]
miR-192	Oncogene	Proliferation, invasion, EMT	SMG-1	[78]
miR-215				
miR-675	Oncogene	Proliferation, invasion, and migration	PITX1	[79]
miRNA-150	Oncogene	Proliferation, migration, EMT	SUFU	[75]
miR-29c-3p	Suppressor	Migration	KIAA1199	[94]
miR-381 miR-489	Suppressor	Growth, migration, and invasion	CUL4B	[95]
miR-375	Suppressor	EMT, migration, invasion	YWHAZ	[97]
miR-338-3p	Suppressor	Proliferation apoptosis	SOX5	[98]
miRNA-27a	Oncogene	Proliferation, invasion	SFRP1	[80]
miR-544a	Oncogene	EMT	CDH1, AXIN2	[81]
miRNA-219-5p	Suppressor	Proliferation, migration, invasion	LRH-1	[99]
MiR-93-5p	Oncogene	Migration, invasion, EMT	AHNAK	[82]
MiR-324-3p	Oncogene	Growth, apoptosis, migration	Smad4	[83]
MiR-142-5p	Suppressor	Migration, invasion	CYR61	[100]
miR-195-5p	Suppressor	Proliferation, apoptosis, and invasion	YAP	[101]

### LncRNAs associated with miRNAs in modulating Wnt/β-catenin signaling

**Fig. 3:**
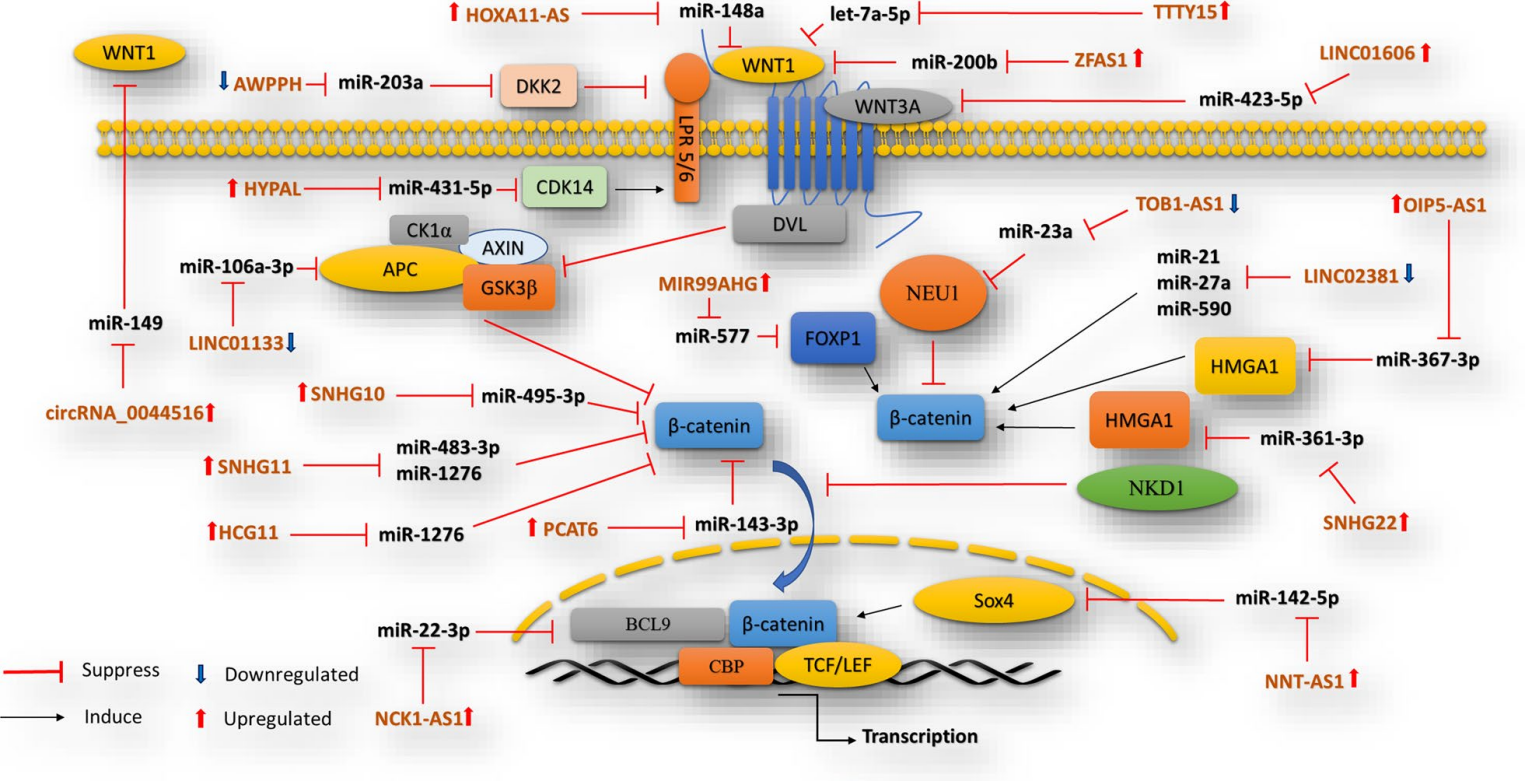
LncRNAs associated with miRNAs in modulating Wnt/β-catenin signaling in GC

Numerous recent studies have established the existence of many types of abnormally expressed lncRNAs in GC [102]. It is described that lncRNAs have a critical function in the initiation and progression of GC, displaying their potential as the main molecular targets for the early diagnosis and treatment of GC [103]. Also, lncRNAs modulate a wide-ranging of signaling pathways, including, Wnt/β-catenin, through competing endogenous RNA (ceRNA) to impact the mRNA expression through sponging miRNAs and modulating their function in GC, as summarized in Fig. 3.

Accumulating studies have evidenced that oncogenic lncRNAs by sequestering miRNAs from their targets stimulate the activation of Wnt/β-catenin signaling. As an oncogenic lncRNA, SNHG11 is highly expressed in GC and contributes to its progression. Mechanically, SNHG11 was illustrated to provoke autophagy through upregulating ATG12 expression and activates β-catenin signaling, leading to an increase in stemness, proliferation, EMT, migration, and invasion in GC cells. SNHG11 upregulated ATG12 and β-catenin expression in post-transcriptional levels via targeting miR-483-3p/miR-1276. SNHG11 also prompted the ubiquitination of GSK-3β by interacting with CUL4A to further activate Wnt/β-catenin pathway [104]. SNHG22 is another oncogenic lncRNA that stimulates the angiogenesis, migration, and invasion of GC cells through modulating miR-361-3p/HMGA1/Wnt/β-catenin signaling [105]. In addition, HCG11 was revealed that is overexpressed in GC tissues and cells. Rescue analysis illustrated that HCG11/miR-1276/CTNNB1 axis modulated the initiation and progression of GC. Tumor forming analysis verified that HCG11 expression was negatively associated with miR-1276 and had a positive relation with CTNNB1 in GC mice models [106]. miR-200b overexpression inhibited the cell cycle progression, and cell proliferation through downregulating Wnt/β-catenin signaling in GC cells. Wnt1 was identified as a target of miR-200b, which in turn is a target of lncRNA ZFAS1. Moreover, Wnt/β-catenin cascade activity prompted via ZFAS1 upregulation was neutralized through overexpressing of miR-200b. Therefore, ZFAS1 was suggested to be involved in GC progression by modulating miR-200b/Wnt/β-catenin signaling pathway [107]. Besides, LINC01606 has been described as a ceRNA participating in Wnt/β-catenin-mediated gastric tumorigenesis. This lncRNA interacts with miR-423-5p and is highly expressed in GC. LINC01606 improved the expression of Wnt3a by sponging miR-423-5p as well as stimulated GC migration/invasion by increasing the activity levels of Wnt/β-catenin signaling [108]. The knockdown of lncRNA NNT-AS1 suppresses the pathogenesis of GC by sponging miR-142-5p and subsequent SOX4-mediated upregulation of Wnt/β-catenin signaling. The suppression of lncRNA NNT-AS1 and/or SOX4 was evidenced to diminish GC cellular proliferation, invasion, and migration as well as enriched apoptosis in GC cells. Basically, lncRNA NNT-AS1 regulated the expression of SOX4 through downregulating miR-142-5p and induced Wnt/β catenin signaling in GC cells [109]. The reports of another study depicted that NCK1-AS1 is an oncogene that functions as a ceRNA and targets miR-22-3p, which directly controls the expression of BCL9. NCK1-AS1 mediated upregulation of BCL9 was also shown to provoke Wnt/β-catenin signaling through gastric tumorigenesis [110]. It is studied that lncRNA prostate cancer-associated transcript 6 (PCAT6) was overexpressed in GC tissues and cells and is related to proteins of cellular proliferation and EMT. It controls the RB/E2F proteins and Wnt/β-catenin signal and miR-15a reverses this activity. Also, miR-15a directly targets PCAT6. Therefore, the suppression of PCAT6 restrained EMT and proliferation by targeting miR-15a via retinoblastoma (RB)/E2F and Wnt/β-catenin pathways in CG cells [111]. LncRNA PCAT6 can activate Wnt/β-catenin pathway. Also, miR-143-3p tumor suppressor was identified as PCAT6 downstream target miRNA, which directly regulates, PRDX5 (peroxiredoxin 5). PCAT6 stimulated stemness and proliferation in GC cells by targeting miR-143-3p and increasing the activity of Wnt signaling mediated by PRDX5 overexpression. Overall, PCAT6 increased tumorigenesis properties via a competing endogenous RNA pattern and activation of that may be a key contribution to treatment of gastrointestinal stromal tumor [112]. Functional investigations stated that lncRNA OIP5-AS1 stimulated proliferation and colony forming whereas prevented apoptosis in GC cells. OIP5-AS1 acted as ceRNA for miR-367-3p. Besides, HMGA2 was identified as a direct target of miR-367-3p that intermediates the impacts of OIP5-AS1 on GC cells. On the other, OIP5-AS1 controlled the activity of Wnt/β-catenin signaling pathways by rescuing HMGA2 expression. Therefore, OIP5-AS1 was suggested to be a stimulator of GC growth by miR-367-3p/HMGA2 axis-mediated regulation of Wnt/β-catenin signaling [113]. LncRNA AWPPH, which is abnormally low-expressed in GC, has been shown to prevent proliferation and invasion in GC cells. MiR-203a was predicted to be a target of AWPPH. Also, miR-203a directly targeted DKK2. The upregulated miR-203a reduced the suppressive effects of AWPPH on GC cell proliferation and invasion. On the contrary, AWPPH upregulation reserved these malignant features by regulating miR-203a/DKK2 axis [114]. MIR99AHG is another oncogenic lncRNA that regulates miR-577/ Forkhead box p1 (FOXP1) axis and by which modulates the ability of GC cells to migrate and invade. It is suggested that upregulated MIR99AHG can sponge miR-577, which results in an enhancement in the expression of FOXP1. Subsequently, the overexpression of FOXP1 induces abnormal acetylation of β-catenin, leading to Wnt signaling aberrant function [115]. Besides, lncRNA HOXA11-AS, as a known oncogenic lncRNA, is involved in GC cell migration and invasion by targeting miR-148a. This miRNA directly targets and downregulates WNT1, which in turn inhibits Wnt/β-catenin signaling in GC. Subsequently, HOXA11-AS overexpression reduced the suppressive effect of miR-148a on WNT1 and increased the activity of Wnt/β-catenin signaling cascade [116]. CTNNB1 has been shown to modulated by SNHG10 lncRNA, which is upregulated through gastric tumorigenesis. Silencing SNHG10 repressed the proliferation, invasion, and migration in GC cells. This phenotype was achieved by rescuing miR-495-3p expression. miR-495-3p is a tumor suppressive miRNA that targets CTNNB1 and inhibits GC progression. Subsequently, the elevated expression of SNHG10 stimulated GC malignant features by modulating miR-495-3p/CTNNB1/Wnt/β-catenin axis [117]. In addition to other oncogenic lncRNA, the overexpressed lncRNA TTTY15 has been also evidenced to increase GC progression through provoking cell proliferation, migration and invasion. TTTY15 upregulation led to Wnt1, β-catenin, and N-cadherin overexpression while prevented the apoptosis and E-cadherin expression in GC. Furthermore, TTTY15 was found to be a ceRNA for let-7a-5p tumor suppressor miRNA. Also, let-7a-5p turned out to directly target Wnt1, and its suppression by TTTY15 rescued Wnt1 expression, which in turn activates Wnt/β-catenin pathway in GC cells [118]. Also, miR-149 was shown to be targeted by circRNA_0044516, and the knockdown of circRNA_0044516 blocked the protein level of β-catenin, and Wnt1 via miR-149. This event suppressed viability and supported apoptosis of GC cells [119].

By the way, despite the oncogenic lncRNAs, some lncRNAs have a suppressive effect on Wnt/β-catenin signaling through interaction with miRNA in GC. A study evidenced that LINC02381 showed a low level of expression in GC samples, indicating its possible tumor suppressive function. By Further investigations, miR-21, miR-27a, and miR-590 were identified to be sponged and downregulated via LINC02381 ceRNA. Furthermore, the overexpression of LINC02381 diminishes Wnt pathway activity. Besides, LINC02381 has a central role in the cell cycle, apoptosis, cell survival, and proliferation of cell lines AGS and MKN45. Taken together, LINC02381, through binding to the candidate microRNAs, may hinder Wnt/β-catenin signal [120]. For more investigation of the role of lncRNAs as a ceRNA in GC, it is reported that LINC01133, as a tumor suppressor, hinders GC development and metastasis. This effect is exerted by sponging miR-106a-3p oncomiR, leading to APC upregulation and consequent deactivation of Wnt/β-catenin cascade [63]. It is shown that HYPAL (Hypoxia Yield Proliferation Associated LncRNA), as an oncogene, functions as a ceRNA that targets miR-431-5p and modulates the expression of Cyclin-dependent kinase 14 (CDK14). Further functional analysis evidenced that Hypoxia-inducible factor-1 α (HIF-1α) induces HYPAL expression in GC cells, which in turn rescues /CDK14 expression by targeting miR-431-5p. Ultimately, this cascade triggers Wnt/β-catenin signaling, prompts cellular proliferation and prevents apoptosis in GC cells [121]. The results of a recent study demonstrated that lncRNA TOB1-AS1 expression was reduced in GC cell lines and tissues. Moreover, the function of TOB1- AS1 suppressing cellular activities was abrogated by NEU1 overexpression. TOB1-AS1 and NEU1 employed their functions through the Wnt/β-catenin signaling. TOB1-AS1 targets miR-23a which in turn directly targets NEU1. Hence, TOB1-AS1/miR-23a/NEU1 axis controlled proliferation, apoptosis, migration, and invasion via Wnt/β-catenin cascade in GC [122]. Table 3 summarized the mentioned interactions.

**Table 3:** The list of lncRNAs involved in Wnt/β-catenin signaling pathway via targeting miRNAs in GC

LncRNA	Oncogene/suppressor	miRNA target	Biological functions	References
TOB1-AS1	Suppressor	miR-23a	Proliferation, apoptosis, invasion, migration	[122]
LINC02381	Suppressor	miR-21, miR-27a, miR-590	Cell cycle, apoptosis, survival proliferation	[120]
SNHG11	Oncogene	miR-483-3p miR-1276	Stemness, proliferation, migration, invasion, EMT	[104]
SNHG22	Oncogene	microRNA-361-3p	Migration, invasion, angiogenesis	[105]
HCG11	Oncogene	miR-1276	Proliferation Migration	[106]
ZFAS1	Oncogene	miR-200b	Cell cycle proliferation	[107]
LINC01606	Oncogene	miR-423-5p	Migrating, Invasion	[108]
NNT-AS1	Oncogene	miR-142-5p	Proliferation, apoptosis, invasion, migration	[109]
NCK1-AS	Oncogene	miR-22-3p	Proliferation, migration, invasion, stemness	[110]
PCAT6	Oncogene	miR-15a	EMT, proliferation	[111]
LINC01133	Suppressor	miR-106a-3p	Development, metastasis	[63]
HYPAL	Oncogene	miR-431-5p	Proliferation, Apoptosis	[121]
PCAT6	Oncogene	miR-143-3p	Stemness, proliferation	[112]
OIP5-AS1	Oncogene	miR-367-3p	Proliferation colony-forming apoptosis	[113]
AWPPH	Suppressor	miR-203a	Proliferation, Invasion	[114]
MIR99AHG	Oncogene	miR-577	Migration, invasion	[115]
HOXA11-AS	Oncogene	MiR-148a	Migration, invasion	[116]
SNHG10	Oncogene	miR-495-3p	Proliferation, invasion, migration	[117]
TTTY15	Oncogene	let-7a-5p	Proliferation, apoptosis migration, invasion	[118]
SNHG22	Oncogene	miR-361-3p	Proliferation, apoptosis, cell cycle, invasion, migration	[105]
circRNA_0044516	Oncogene	miR-149	Cell viability and apoptosis	[119]

### LncRNAs regulate Wnt/ β-catenin signaling by affecting molecular components

LncRNAs also participate in the modulation of Wnt/β-catenin signaling cascade directly or by affecting molecular components rather than miRNAs in GC. Considering their targets, these lncRNAs are also divided into two groups of oncogenic and tumor suppressor lncRNAs. As a known oncogenic lncRNA, ZEB2-AS1, participates in the pathogenesis of GC by induction of β-catenin expression through ZEB2 overexpression [123]. Also, upregulated LncRNA SNHG16 (small nucleolar RNA host gene 16) has been illustrated to increase EMT and invasion via downregulating DKK3 tumor suppressor in GC cells, directly or indirectly [124]. The lncRNA ultraconserved region (UC) 0.145 was also shown to modulate epigenetic regulation of DKK1, as another repressor of Wnt/β catenin cascade, in GC cells. It has been depicted that UC.145 induces DKK1 hypermethylation in cooperation with EZH2 and participates in Wnt/β-catenin signaling. Moreover, the interplay between UC.145 and another lncRNA, known as PRKG1-AS1, explicated a synergistic impact on Wnt cascade by downregulating DKK1 expression. The suppression of UC.145 prompted cell apoptosis and prevented the growth, invasion, migratory ability, and colony formation in GC cells [125]. Also, high expression of LINC01503 in GC tissues and cells was shown to be correlated to the activity of Wnt/β-catenin pathway. The blockage of LINC01503 expression repressed proliferation and invasion of GC cells. This effect was aligned with a decrease in expression levels of β-catenin, cyclin D1, and c-myc. On the contrary, the elevated expression of LINC01503 enhanced GC cell proliferation and metastasis by modulating Wnt/β-catenin pathway [126]. Besides, a study implied that lncRNA ZFAS1 is another oncogenic lncRNA, and its suppression could prevent gastric tumorigenesis and chemoresistance by downregulating β-catenin expression [127]. The suppression of TP73-AS1, as an oncogene, was revealed to significantly prevent proliferation, invasion, and colony forming in GC cells. Moreover, the downregulated TP73-AS1 blocked the expression of TCF4 and β-catenin, repressing WNT/β-catenin cascade in GC cells [128]. Additionally, it was illustrated that VIM Antisense RNA 1 (VIM-AS1) was overexpressed in GC cells and tissues, corresponding to the enhanced AGS and HGC-27 GC cell proliferation, migration, and invasion. The suppression of VIM-AS1 notably enhanced Bax, cleaved caspase-3, and E-cadherin protein expression, but reduced the expression of N-cadherin, Bcl-2, vimentin, (MMP)-2/9, β-catenin, C-myc, cyclin D, and FZD1 in protein levels. Accumulatively, VIM-AS1 can enhance tumor progression by regulating FDZ1 expression and activation of Wnt/β-catenin cascade in GC [129]. DLGAP1-AS2 has been recently described as an oncogenic lncRNA showing high levels of expression in GC patients. The suppression of DLGAP1-AS2 limited proliferation, invasion, and migration in GC cell lines through downregulation of Wnt1 expression. To be more clarified, DLGAP1-AS2 was directly engaged with Six3 transcriptional repressor, leading to the inhibition of Six3 binding to the promoter regions of Wnt1 gene. Subsequently, reduced Wnt1 expression hampered the activity of Wnt/β-catenin signaling. Therefore, targeting DLGAP1-AS2/Six3/Wnt1/β-catenin axis may be suggested as an effective strategy to develop novel s therapeutic approaches for GC [130]. In addition, GSK-3β, as an important suppressive component of Wnt/β-catenin signaling, has been reported to be regulated by lncRNA SNHG20. SNHG20 upregulation increased GC malignant features by modulating the GSK-3β/β-catenin pathway and blocking p21 expression [131].

In addition to oncogenic lncRNAs, there are some tumor suppressor lncRNAs that also regulate Wnt/ β-catenin signaling by affecting molecular components. It is found that suppression of LOC285194, which showed low expression levels in GC tissues and cell lines, stimulated GC progression via prompting Wnt signaling activity. LOC285194 was illustrated to directly regulate β‐catenin and GSK‐3 β protein levels [132]. Another study indicated that the expression of β-catenin, Wnt2b, N-cadherin, cyclinD1, vimentin, and snail was upregulated, whereas the ENST00000434223 and E-cadherin expression levels were downregulated in GC tissues. LncRNA-ENST00000434223 was also reported to suppress tumor progression in GC cells by reducing Wnt/β-catenin signaling activity [133]. It was also reported that LINC01314, which targets KLK4 (Kallikrein Related Peptidase 4) expression, is downregulated through gastric tumorigenesis. The upregulation of LINC01314 or silencing of KLK4, an inducer of β-catenin signaling, resulted in the inhibition of GC cell migration and invasion, corresponding to reduced expression levels of β-catenin, Wnt-1, N-cadherin, cyclin D1, whereas improved E-cadherin expression. LINC01314 was also suggested as another tumor suppressor that blocks the activity of Wnt/β-catenin signaling by downregulating KLK4, suppressing GC invasion, migration, and angiogenesis [134].

## Conclusion and perspective

The Wnt/β-catenin signaling pathway is considered a well-recognized oncogenic pathway in a wide array of human cancers, including GC, that is involved in cancer initiation and progression. Although a large number of studies assessed underlying molecular mechanisms related to Wnt/β-catenin mediated gastric tumorigenesis, and many therapeutic methods were investigated for the suppression of this oncogenic pathway, elimination of some drawbacks in therapies targeting Wnt/β-catenin signaling, such as resistance or unresponsiveness, need additional investigations. Accordingly, this review endeavored to elucidate the downstream and upstream regulators of Wnt/β-catenin signaling in GC cells to facilitate the understanding of pathways involved in the progression of this malignancy in terms of its treatment and diagnosis. As mentioned in this study, various miRNAs and lncRNAs, as dual role regulators, could directly or indirectly activate or suppress Wnt/β-catenin pathway, getting involved in GC progression. More importantly, a wide range of miRNAs, including miR-200b, which function as a tumor suppressor and targets the main component of Wnt/β-catenin signaling, such WNT1, are blocked by ceRNA functioning of lncRNAs, hereby oncogenic lncRNA ZFAS1, resulting in increased gastric cancer cell growth and proliferation. On the contrary, deregulated expression of some tumor suppressive lncRNAs, as an example LINC01133, leads to hyperactivity of some oncomiRs, such as miR-106a-3p targeting pivotal suppressors of Wnt/β-catenin signaling like APC, that contributes to gastric cancer development and metastasis. Consequently, the interplay between these ncRNAs and Wnt/β-catenin signaling seems to be a crucial mechanism in regulating gastric tumorigenesis. Therefore, targeting lncRNAs/miRNAs/β-catenin axis may be a promising alternative for other targeted therapies based on Wnt/β-catenin signaling. For instance, using of the RNA interference (RNAi) technique targeting ncRNAs has been evidenced to increase the hope of the successful treatment of GC patients by modulating Wnt/β-catenin signaling. These methods are also restricted by some obstacles such as low bioavailability and delivery into cancer cells. Nevertheless, applying novel methods using nanoparticles ensuring high cellular uptake through encapsulation of these regulatory RNA molecules and their site-specific delivery may pave GC therapy.

## Data Availability

Not applicable.

## Abbreviations

GC: Gastric cancer
ncRNA: Non-coding RNA
lncRNA: Long non-coding RNA
miR: MicroRNA
siRNA: Small interfering RNAs
piRNA: Piwi-interacting RNAs
tRNA: Transfer RNAs
rRNA: Ribosomal RNAs
circRNA: Circular RNAs
snoRNA: Small nucleolar RNA
tRF: TRNA-derived small RNA
WNT: Wingless-related integration site
MMTV: Mouse mammary tumor virus
CK1: Casein kinase 1
GSK-3α/β: Glycogen synthase kinase 3α/β
APC: Adenomatous polyposis coli
AXIN1: Axis inhibition protein 1
β-TrCP: β-Transducin repeat-containing protein
TLE: Transducing-like enhancer protein
TCF/LEF: T-cell factor/lymphoid enhancer-binding factor
HDAC: Histone deacetylases
FZD: Frizzled
LRP: Low density lipoprotein receptor–related protein
Dvl: Dishevelled
CBP: CREB binding protein
BCL9: B-cell lymphoma 9
BRG1: Brahma-related gene-1
PCP: Planar cell polarity
ROR: Retinoic acid-related orphan receptors
DAAM1: Dvl associated activator of morphogenesis 1
ROCK: Rho kinase
JNK: C-Jun NH2-terminal kinases
ATF2: Activation of transcription factor 2
Vangl: Van Gogh-like
CTNNB1: Catenin beta 1
RNF43: Ring finger protein 43
DKK: Dickkopf WNT signaling pathway inhibitor
SFRP: Secreted frizzled related protein
NKD: Naked cuticle
EMT: Epithelial–mesenchymal transition
WIF: Wnt inhibitory factor
EBV: Epstein-Barr virus
MMP: Matrix metallopeptidase
SUFU: Suppressor of fused homolog
PTEN: Phosphatase and tensin homolog
DIXDC1: Dishevelled–Axin domain containing 1
EphA2: Ephrin type-A receptor 2
HMGA: High mobility group protein
PRRX: Paired related homeobox
PI3K/AKT: Phosphatidylinositol 3-kinase/protein kinase B
TRIM24: Tripartite motif-containing 24
GPRIN3: GPRIN family member 3
MMTV: Chromobox
HIF: Hypoxia-inducible factor
ZEB1: Zinc finger E-box binding homeobox 1
SMG: SMG nonsense mediated mRNA decay associated PI3K related kinase
EGFR: Epidermal growth factor receptor
FGFR: Fibroblast growth factor receptor
PTP4A3: Protein tyrosine phosphatase 4A3
CUL4B: Cullin 4B
YWHAZ: Tyrosine 3 monooxygenase/tryptophan 5-monooxygenase activation protein zeta
SOX5: SRY-box transcription factor 5
VIM: Vimentin
CDH1: Cadherin 1
SNAIL: Zinc finger protein SNAI1
CYR61: Cysteine-rich angiogenic inducer 61
FBN1: Fibrillin 1
YAP: Yes-associated protein
LRH1: Liver receptor homolog 1
ATG12: Autophagy related 12
PCAT6: Prostate cancer-associated transcript 6
RB: Retinoblastoma
CDK14: Cyclin-dependent kinase 14
HYPAL: Hypoxia yield proliferation associated LncRNA
PRDX5: Peroxiredoxin 5
FOXP1: Forkhead box p1
SNHG16: Small nucleolar RNA host gene 16
AS1: Antisense RNA 1
TOB1: Transducer of ERBB2, 1
HCG11: HLA complex group 11
NNT: Nicotinamide nucleotide transhydrogenase
NCK1: Non-catalytic region of tyrosine kinase adaptor protein 1
OIP5: Opa interacting protein 5
TTTY15: Testis-specific transcript, Y-linked 15
